# Oral Health Status of Syrian Children in the Refugee Center of Melilla, Spain

**DOI:** 10.1155/2018/2637508

**Published:** 2018-03-18

**Authors:** Sabrina Gonçalves Riatto, Javier Montero, David Ribas Pérez, Antonio Castaño-Séiquer, Abraham Dib

**Affiliations:** ^1^Faculty of Dentistry, IESP, Joao Pessoa, PB, Brazil; ^2^Department of Stomatology, University of Seville, Seville, Spain; ^3^Department of Surgery, University of Salamanca, Salamanca, Spain

## Abstract

**Introduction:**

Little is known about the state of oral health among immigrants from conflict zones, such as the refugee children from the Syrian Civil War.

**Aim:**

To determine the oral health status of Syrian immigrant children refugee at the Center for Temporary Stay of Immigrants in Melilla to plan prevention and care programs.

**Design:**

Using the criteria set by the World Health Organization, an exploration of the oral cavity of all Syrian children aged 5–13 living at that center was conducted in May 2015. All subjects were clinically evaluated by a calibrated and standardized examiner, accompanied by a dentist who registered the clinical variables, and translators. The sociodemographic and clinical variables were analyzed through a descriptive and analytical study, respectively.

**Results:**

The prevalence of caries in both the permanent and deciduous dentition was 75% and 50% in 6- and 12-year-olds, respectively. The dft was 3.2 ± 2.9 in 6-year-old children. At 12 years old, the DMFT was 1.6 ± 2.6 teeth, the DMFM was 1.1 ± 1.7 teeth, the SiC was 3.2, and the IR was 5%. Eighty-six percent of the examined sextants were periodontally healthy.

**Conclusions:**

The prevalence of caries was high in the sample population studied, confirming the need for a comprehensive primary oral health care program.

## 1. Introduction

According to the World Health Organization (WHO), oral disease is one of the most prevalent health care problems [1]. Dental caries are the most frequent form of oral disease and are considered a public health problem affecting both children and adults worldwide and often lead to a significant reduction in the quality of life of individuals and communities [2, 3]. Periodontal disease also significantly contributes to global oral disease, affecting all age groups [4]. The WHO regularly monitors the health status of the main age groups, with the intention of promoting better oral health. Recent studies have shown that immigrant populations are more affected by dental caries than native populations [5–8]. However, very little is known about the state of oral health among immigrants originating from conflict zones.

In 2011, a civil war began in Syria, which has caused one of the largest refugee exoduses of recent history [9]. According to the United Nations High Commissioner for Refugees (UNHCR available at http://data.unhcr.org/syrianrefugees/regional.php), as of May 2016, almost 5 million Syrian refugees have been registered since the beginning of the armed struggle. The vast majority of refugees are located in Turkey, Lebanon, Jordan, Iraq, Egypt, and North Africa, and 53% of them are aged younger than 18 years. Although this number of people is low (10%) compared to the number of refugees originating from other neighboring countries in conflict, the amount of Syrians seeking asylum in Europe is still rising. Nevertheless, according to the abovementioned website, applications for international protection have been registered in 37 European countries: 62% in Serbia and Germany; 27% in Sweden, Hungary, Austria, Netherlands, and Denmark; and 11% in all of the other countries, including Spain, with almost 9000 applications. The Syrian immigrants arrive to Spain by the so-called “southern route” where they hope to find in the Spanish city of Melilla, located in the north of the African continent, a gateway to Europe.

The extreme situation in which people live along the border between Melilla and the Moroccan city of Nador has resulted in the concentration of approximately 2000 refugees in the Center for Temporary Stay of Immigrants (CETI), which has a maximum capacity of accommodating 480 individuals. Among this population are around 300 children living in overcrowded conditions and exposed to general health risks, as affirmed by the Spanish Government. This community faces economic, cultural, and linguistic barriers that hinder their access to oral health services deployed in the city. To address this problem, proper planning of oral health services is needed to be able to evaluate the current status of oral health and to identify the main type of treatments needed in order to set priorities and plan the different preventive and curative strategies.

This study sought to explore the state of oral health in the population of Syrian immigrant refugee children living at the CETI in Melilla, Spain, which have otherwise not received help to control oral disease.

## 2. Materials and Methods

In May 2015, refugee Syrian children living at the Center for Temporary Stay of Immigrants (CETI) in Melilla (Spain) were evaluated, following the guidelines recommended by the WHO [10]. Of the 300 children at the CETI, only those aged 5 to 13 years were included within the study.

This work was approved by the Ethics Committee of Andalusia (approval protocol number: 057575944–0905N14) and authorized by the CETI of Melilla, and signed consent was obtained from the individuals responsible for each child participating in the study.

The oral examination of each participant was carried out by a dentist (first author) who had been previously trained in the basic methods and standardized diagnostic criteria outlined by the WHO [10] for the evaluation of different oral conditions. Previous to the start of the study, the examiner was tested using calibration tests of intraexaminer concordance and showed excellent results that supported their suitability to carry out the work. The results obtained were in accordance with test kappa = 0.90, which represents a level of agreement of “almost complete agreement,” according to the scale of Landis and Koch [11].

During clinical examinations, the examiner was next to the patient accommodated in a reclining chair. Natural and artificial lighting was supplemented using a portable front focus white light lamp of 100 W (Kopfleuchte Heine SL 350 Optotechnik, Germany). The clinical variables were measured using a basic disposable plane oral mirror and a WHO periodontal probe.

The exploration was attended by a dentist who registered the clinical variables and a bilingual organizer assistant (Arabic and Spanish speaker) who completed the general descriptive information about each child. Arabic translators were present to facilitate communication between the review team with children and/or parents who did not speak Spanish. One of the translators observed while the questionnaire/form was being filled in to ensure that no mistakes were made in recording the information. The average scan time for each child was around 4-5 minutes.

The oral health assessment questionnaire/form was developed based on the model proposed by the WHO [10] in which sociodemographic variables (age, gender, religious beliefs, and ethnicity) and clinical variables (healthy teeth, decayed teeth, missing or filled teeth, healthy sextants, bleeding sextants, or sextants with calculus) were registered.

The following indexes of caries history were calculated: DMFT (mean of decayed, missing, and filled permanent teeth) [12], dft (mean of decayed and filled primary teeth) [13], and DMFM (mean of decayed, missing, and filled first permanent molars); significant caries index (SiC), which reflects the mean of decayed teeth in the third of the population with the highest decayed teeth component in the DMFT [14], and the index of restoration (IR) for the filled teeth.

The global DMFT was calculated by computing the decayed, missing, or filled teeth independent of the type of dentition, since the dentition in this age group (5–13 years) was mostly mixed.

The SiC was calculated using the program developed by the WHO available at http://www.whocollab.od.mah.se/expl/siccalculation.xls.

Periodontal status was registered using the community periodontal index (CPI) recommended by the WHO for children under 15 years [10]. The mean number of sextants with healthy periodontal tissues, bleeding teeth, and teeth with calculus was calculated to describe the periodontal health.

The statistical analysis of the data was carried out using the program SPSS Statistics 20. The statistics carried out on the descriptive data used means, standard deviations (SD), number of subjects (*n*), and percentages. In addition, comparisons of the quantitative variables between two or more groups were performed using Student's *t*-test and ANOVA with Bonferroni correction, respectively. The influence of age on the state of the oral health of the children examined was analyzed by the Pearson correlation.

## 3. Results

According to Table 1, which shows the sociodemographic description, most children were between the ages of 8 and 10 years (42%), followed by one group of 5- to 7-year-olds (33%) and one group of 11- to 13-year-olds (25%). Of the total sample, 57% were males and mainly of Arab origin (64%), where 36% were of Caucasians. The sample had a mean age of 9.0 ± 2.2 years.

Table 2 depicts the oral health status of the sample and the comparisons regarding several indexes among the three age groups after the analysis of variance (ANOVA). When the focus was directed towards the estimation of the global caries (in both deciduous and permanent dentition), it was found that prevalence of caries (59%) was lower in the older children (11–13 years) than in the children of the younger age groups (over 74%). However, caries in the permanent dentition increase with age, and the prevalence in the deciduous dentition decreases with aging. Furthermore, it was observed that, in the oldest children, there were fewer active caries (decayed teeth + teeth filled with caries) and therefore a greater number of decay-free teeth (healthy teeth + filled teeth + with crown + fractured). Regarding the indicators indexes of caries history, an increase was observed in the DMFT, DMFM, and IR, according to the age, whereas the dft decreased in the oldest group of children. After analyzing the sextants using the different CPI codes, the majority of the sextants were found to be healthy in all of the age groups studied. A SiC of 3.2 ± 3.0 decayed teeth was obtained for the 12-year-old children. The index of restoration was zero in the majority of the children of every subgroup (≥90%), reflecting that dental caries were mostly untreated.

Table 3 shows the influence of ethnicity and gender in the oral health of Syrian children after comparison with Student's *t*-test. Significant differences were observed in the number of healthy and decayed teeth, with the best oral health status within the non-Arab group. Additionally, it was established that gender had no significant influence on the oral health status of the whole sample after analyzing the corresponding data. However, when analyzing the various age groups separately, significant differences in the number of decayed teeth, global DMFT, and sextants with bleeding were found. The 5- to 7-year-old girls had higher values than the boys of the same age and, therefore, had a worse state of general oral health. In contrast, the group of 11- to 13-year-old boys had significant worse oral health than the girls.

**Figure 1:**
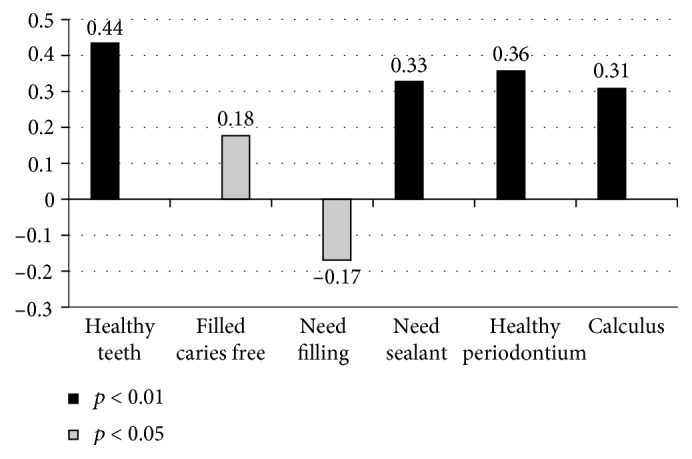
Influence of age on the oral health of Syrian children in the CETI of Melilla. Pearson correlation coefficients between age and oral health variables.

The analysis of the influence of age on the oral health status by the Pearson linear correlation is displayed in Figure 1. Age was found to be positively correlated with the presence of healthy nonrestored teeth (*r*: 0.44; *p* < 0.01) and consequently with the number of first molars needing sealants (*r*: 0.33; *p* < 0.01) but also with the number of filled teeth without caries (*r*: 0.18; *p* < 0.05). Similarly, age was inversely correlated with the number of decayed teeth needing fillings. In periodontal terms, age was directly related with both the presence of healthy periodontal sextants (*r*: 0.36; *p* < 0.01), but also with the presence of sextants with calculus (*r*: 0.31; *p* < 0.01).

These data demonstrate that within this short age range (5–13 years) which comprises the eruption period of the permanent teeth, the older children in the sample population have more healthy standing teeth with optimal health (crown and periodontium). However, paradoxically, an increase in the presence of calculus is also observed around the gingival margin of the targeted teeth for the CPI.

## 4. Discussion

The aim of this study was to register the oral health status of Syrian refugee children living in the CETI in Melilla, Spain, during 2015, using the methodological dossier established by the WHO [10]. All 5- to 13-year-old Syrians were examined and, for logistical reasons, were divided into three age cohorts according to the activities that were taking place within the center. In addition, a microanalysis of the 6- and 12-year-old children was conducted with the intention of comparing the oral health status of this group with other paediatric populations. In order to compare the results, reference studies involving immigrants in Spain [5], North Africans [2, 15–21], Syrians [22–24], and Middle East children [18, 25–27] were used.

It can be expected that the immigrants from war zones have very poor oral health. However, paradoxically, the results obtained were not so negative and may be attributed to the average socioeconomic level of the population studied, where the parents of the children had jobs and incomes, before they were affected by conflict, therefore coming from wealthier families. Also, a low global index of restoration (5.0 ± 15.8% teeth at 12 years old), in accordance with the goals of oral health (IR ≥ 60%) proposed by Spain for children of this age by 2015 [28], was observed. The IR is considered to be an indicator of the level of dental care received by the target population and may be linked to its economic power, as well as the degree of information and access to health services. This fact may be attributed to the young age of the children at the start of the armed struggle, an event which drastically changed the routine of these families, where reparative oral health treatments become a total nonpriority [29]. In any case, immigrant children are expected to climb down the levels of oral health of the host countries [30].

The high prevalence of dental caries in children is linked to the habits and cultural factors of each population [31], which include eating habits (unhealthy or high sugar), poor dental hygiene, lack of dental care services and prevention programs [32], as well as the widespread use of fluorides from different sources [33].

In many Middle Eastern countries, the prevalence and severity of dental caries is one of the main problems of oral health due to the deficiency of prevention programs [34]. In regard to African countries, the incidence of caries is less prevalent or severe than in developed countries. However, this situation is changing, and the incidence of caries is expected to increase as a result of a higher consumption of sugars, the popularity of western food, and also to the difficulties to access dental care [18].

**Figure 2:**
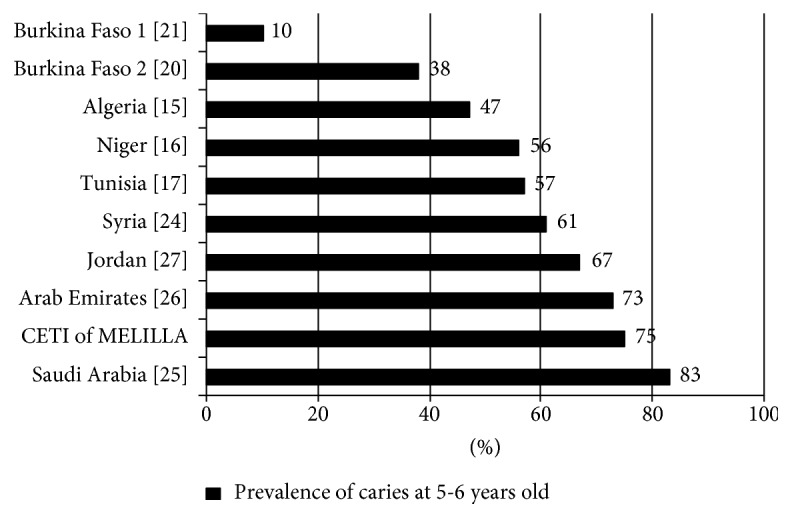
Prevalence of caries in 5- to 6-year-old children in some countries within Africa and in the Middle East, as well as in the CETI of Melilla.

Figure 2 shows a higher prevalence of caries in 5- to 6-year-olds in the Middle Eastern countries than in African countries. Also, other studies involving children of these ages showed that the countries with the highest prevalence of caries were Saudi Arabia [25], Arab Emirates [26], and Jordan [27]; countries with an intermediate prevalence of caries were Syria [24], Tunisia [17], and Niger [16]; and countries with the lowest prevalence of caries were Algeria [15] and Burkina Faso [20, 21].

**Figure 3:**
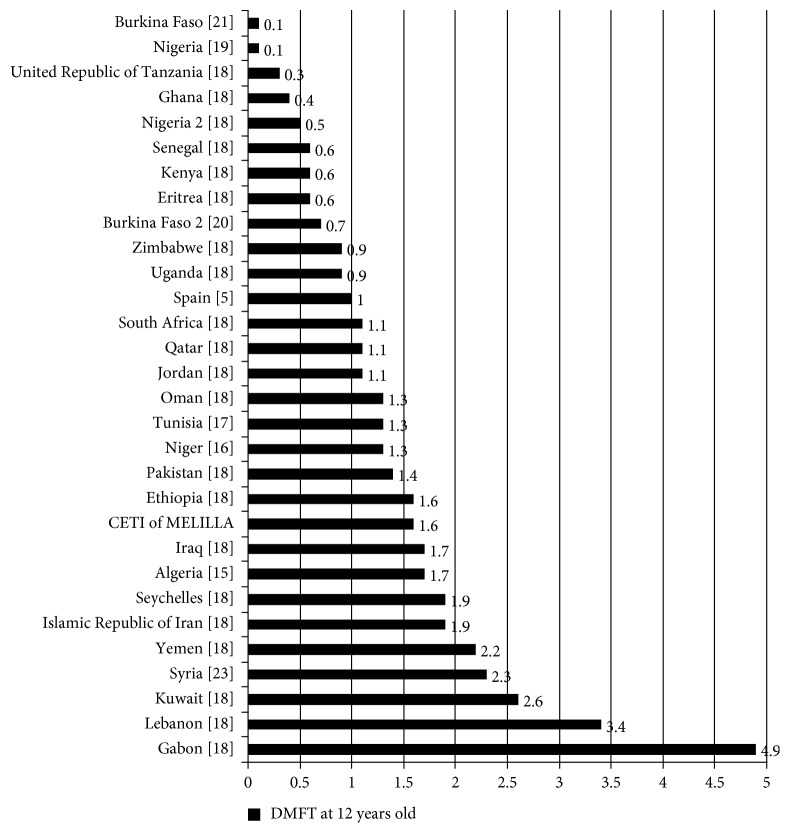
DMFT of 12-year-olds in some countries within the Middle East and Africa, as well as in Spain and in the CETI of Melilla.

According to the WHO [10], DMFT values are considered: very low up to 1.1 teeth; low between 1.2 and 2.6 teeth; moderate between 2.7 and 4.4 teeth; high between 4.5 and 6.5 teeth; and very high up to 6.5 teeth.  Figure 3 exhibits the results of studies involving 12-year-old children from different countries, where it can be observed that very low DMFT values were found in Burkina Faso [21], Nigeria [19], United Republic of Tanzania [18], Ghana [18], Nigeria 2 [18], Senegal [18], Kenya [18], Eritrea [18], Burkina Faso 2 [20], Zimbabwe [18], Uganda [18], Spain [5], South Africa [18], Qatar [18], and Jordan [18]. Low DMFT values were found in Oman [18], Tunisia [17], Niger [16], Pakistan [18], Ethiopia [18], Iraq [18], Algeria [15], Seychelles [18], Islamic Republic of Iran [18], Yemen [18], Syria [23], and Kuwait [18]. A moderate DMFT value was found in Lebanon [18], and a high DMFT value was found in Gabon [18].

Also, the result of the low level of dental caries registered by Almerich-Silla et al. [15] in children studied in the southern region of Algeria (Sahara) was attributed to the exposure of the population to high concentrations of fluoride in the drinking water.

The 12-year-old Syrian children studied by Beiruti et al. [23] presented a DMFT of 2.3 teeth, which is higher than the findings of this study for children of the same age (1.6 ± 2.6 teeth). However, we need to be cautious in interpreting this data because our sample only included ten 12-year-olds. Furthermore, we found a greater DMFT in the children living at the CETI in Melilla than in Spain—although lower than in Syria.

Among African populations, conditions of oral hygiene are generally deficient, and the methods used for oral cleaning can be unconventional (such as using sticks or sponges gum, banana stems with carbon powder, plants leaves with ash used with cotton, cloth, or fingers), and lead to plaque formation and consequently calculus formation, even at early ages [35]. Furthermore, in Syria the use of conventional oral hygiene methods was reported, and Dashash et al. [24] also found a strong association between the oral hygiene habits of the parents and the children, where 53% of the children with poor oral hygiene came from families who did not report having good oral hygiene habits. Beiruti et al. [22] reported that 26% of Syrian adolescents have healthy periodontium. Some years later, the same authors in another study reported that 94% of the adolescents examined had an accumulation of dental plaque due to poor oral hygiene [23]. These various studies help to confirm that the population examined in the CETI of Melilla had a better level of periodontal health for all age groups (86% of the sextants were healthy, 12% were not evaluable, and only 3% had bleeding and/or calculus and 8% of the 12-year-old children had bleeding and/or calculus).

The main limitation of this study was that no information regarding the frequency and techniques of oral hygiene, the use of fluoride toothpaste or mouth rinses, or the diet of the Syrian children before entering into the CETI was collected. In any case, each child in the CETI had access to an oral hygiene kit consisting of a toothbrush and toothpaste and was instructed to brush their teeth after each meal and before to continue educational activities aimed at them. Future studies need to be conducted that accurately assess these behavioral factors that are jointly responsible for the inherent risk of oral diseases, considered desirable to know what the oral health circumstances and maintenance before being housed as immigrants.

## 5. Conclusion

The oral health status of Syrian children refugee in the CETI of Melilla showed a high prevalence of caries, especially, in primary teeth as well as in permanent teeth (affecting 75% in children under 10 years). The index of restoration observed was very low (5%); however, the periodontal health was satisfactory where most of the sextants were healthy (86%).

## Figures and Tables

**Table 1:** Sociodemographic description.

Sociodemographic variables		Age groups							
		All ages		5–7 years old		8–10 years old		11–13 years old	
		%	*n*	%	*n*	%	*n*	%	*n*
		100	156	33	51	42	66	25	39
Gender	Male	57	89	57	29	44	37	59	23
	Female	43	67	43	22	56	29	41	16
Ethnicity	Caucasian	36	56	37	19	35	23	36	14
	Arab	64	100	63	32	65	43	64	25

**Table 2:** Oral health status within the distinct age groups.

Variables	Age groups					
	5–7 yrs (*n*=51)	8–10 yrs (*n*=66)	11–13 yrs (*n*=39)	6 yrs (*n*=28)	12 yrs (*n*=10)	5–13 yrs (*n*=156)
Global caries prevalence (DMFT or dft > 1) (% of subjects)	74.5	74.2	59.0	75.0	50.0	70.5
Caries prevalence in permanent dentition (% of subjects)	7.8	34.8	41.0	7.1	50.0	27.6
Caries prevalence in deciduous dentition (% of subjects)	72.5	60.6	25.6	75.0	0.0	55.8
DMFT (mean ± SD)^††a,b,c^	0.1 ± 0.4	0.7 ± 1.1	1.8 ± 3.1	0.1 ± 0.5	1.6 ± 2.6	0.8 ± 1.8
Decayed permanent teeth (mean ± SD)^††a,b,c^	0.1 ± 0.4	0.7 ± 1.0	1.5 ± 2.8	0.1 ± 0.5	1.4 ± 2.5	0.7 ± 1.7
Missing permanent teeth (mean ± SD)	0.0 ± 0.0	0.0 ± 0.1	0.1 ± 0.2	0.0 ± 0.0	0.0 ± 0.0	0.0 ± 0.1
Filled permanent teeth (mean ± SD)^†(a/b),c^	0.0 ± 0.0	0.0 ± 0.0	0.2 ± 0.8	0.0 ± 0.0	0.2 ± 0.6	0.1 ± 0.4
IR (mean ± SD)	0.6 ± 4.7	2.8 ± 13.6	4.2 ± 14.2	0.0 ± 0.0	5.0 ± 15.8	2.5 ± 11.6
DMFM (mean ± SD)^††a,(b/c)^	0.1 ± 0.4	0.6 ± 0.9	0.9 ± 1.4	0.1 ± 0.5	1.1 ± 1.7	0.5 ± 1.0
dft (mean ± SD)^††a,c^	3.2 ± 3.6	2.2 ± 2.5	0.9 ± 2.2	3.2 ± 2.9	0.0 ± 0.0	2.2 ± 2.9
CPI 0 = healthy sextants (mean ± SD)^††a,(b/c)^	4.0 ± 2.5	5.8 ± 0.6	5.5 ± 1.0	2.8 ± 2.7	5.4 ± 1.4	5.1 ± 1.8
CPI 1 = bleeding sextants (mean ± SD)	0.0 ± 0.0	0.0 ± 0.4	0.1 ± 0.4	0.0 ± 0.0	0.0 ± 0.0	0.1 ± 0.3
CPI 2 = sextants with calculus (mean ± SD)^††(a/b,c)^	0.0 ± 0.0	0.0 ± 0.3	0.4 ± 0.9	0.0 ± 0.0	0.6 ± 1.4	0.1 ± 0.5

DMFT: mean of decayed, missing, and filled permanent teeth; IR: index of restoration for the filled teeth (permanent and deciduous); DMFM: mean of decayed, missing, and filled first permanent molars; dft: mean of decayed and filled primary teeth; CPI: community periodontal index. ^†^Statistically significant intergroup comparisons according to ANOVA test (*p* < 0.05). ^††^Statistically significant intergroup comparisons according to ANOVA test (*p* < 0.01). ^a,b,c^The letters beside the symbol “^†^” indicate the subgroups (a = 5–7 yrs; b = 8–10 yrs; and c = 11–13 yrs) that are statistically different after post hoc Bonferroni correction.

**Table 3:** Influence of ethnicity and gender in the oral health status of Syrian children in the CETI of Melilla (*n*=156).

Sociodemographic variables			Oral health variables					
			Healthy teeth	Decayed teeth	Global DMFT	Global IR	Healthy sextants	Bleeding sextants
Ethnicity (mean ± SD)	Caucasian		21.3 ± 3.9^∗^	2.3 ± 2.9^∗^	2.7 ± 3.6	1.8 ± 8.2	5.2 ± 1.6	0.0 ± 0.3
	Arab		20.0 ± 4.3^∗^	3.2 ± 3.3^∗^	3.5 ± 3.6	2.8 ± 13.3	5.1 ± 1.9	0.0 ± 0.3
Gender (mean ± SD)	All the sample	Male	20.5 ± 3.7	2.9 ± 3.0	3.1 ± 3.1	2.2 ± 12.6	5.0 ± 1.9	0.1 ± 0.4
		Female	20.4 ± 4.8	2.8 ± 3.5	3.3 ± 4.1	2.7 ± 10.3	5.4 ± 1.5	0.0 ± 0.0
	5–7 years old	Male	19.2 ± 3.6	2.5 ± 2.9^∗^	2.6 ± 3.0^∗^	0.0 ± 0.0	3.6 ± 2.7	0.0 ± 0.0
		Female	17.9 ± 5.0	4.4 ± 4.4^∗^	4.9 ± 5.1^∗^	1.5 ± 7.1	4.5 ± 2.3	0.0 ± 0.0
	8–10 years old	Male	20.4 ± 2.8	2.9 ± 2.3	3.2 ± 2.5	3.6 ± 17.2	5.8 ± 0.7	0.0 ± 0.5
		Female	20.1 ± 4.1	2.7 ± 3.1	3.2 ± 3.8	1.7 ± 6.8	5.9 ± 0.4	0.0 ± 0.0
	11–13 years old	Male	22.1 ± 4.6^∗^	3.3 ± 3.9^∗^	3.7 ± 4.1^∗^	2.8 ± 11.9	5.4 ± 1.2	0.2 ± 0.5^∗^
		Female	24.5 ± 2.5^∗^	0.9 ± 1.6^∗^	1.3 ± 1.9^∗^	6.3 ± 17.1	5.7 ± 0.7	0.0 ± 0.0

^∗^Significant difference with *p* < 0.05 between both groups after Student's *t*-test.

## References

[1] WHO (World Health Organization) (2000). *Global Oral Health Data Bank*.

[2] Bourgeois D. M., Llodra J. C. (2014). Global burden of dental condition among children in nine countries participating in an international oral health promotion programme, 2012-2013. *International Dental Journal*.

[3] Sheiham A. (2005). Oral health, general health and quality of life. *Bulletin of the World Health Organization*.

[4] Petersen P. E., Bourgeois D., Ogawa H., Estupinan-day S., Ndiaye C. (2005). The global burden of oral diseases and risks to oral health. *Bulletin of the World Health Organization*.

[5] Almerich-Silla J. M., Montiel-Company J. M. (2007). Influence of immigration and other factors on caries in 12- and 15-yr-old children. *European Journal of Oral Sciences*.

[6] Cvikl B., Haubenberger-Praml G., Drabo P. (2014). Migration background is associated with caries in Viennese school children, even if parents have received a higher education. *BMC Oral Health*.

[7] Riggs E., Gussy M., Gibbs L. (2014). Assessing the cultural competence of oral health research conducted with migrant children. *Community Dentistry and Oral Epidemiology*.

[8] Chen C. C., Chiou S. J., Ting C. C. (2014). Immigrant-native differences in caries-related knowledge, attitude, and oral health behaviors: a cross-sectional study in Taiwan. *BMC Oral Health*.

[9] Bahelah R., Fouad F. M., Coutts A., Wilcox M., Maziak W. (2014). Syria: health in a country undergoing tragic transition. *International Journal of Public Health*.

[10] WHO (1997). *Oral Health Surveys: Basic Methods*.

[11] Landis J. R., Koch G. G. (1977). The measurement of observer agreement for categorical data. *Biometrics*.

[12] Klein H., Palmer C. E. (1938). *Dental Caries in the American Indian Children*.

[13] Gruebbel A. O. (1944). A measurement of dental caries prevalence and treatment service for deciduous teeth. *Journal of Dental Research*.

[14] Bratthall D. (2000). Introducing the Significant Caries Index together with a proposal for a new global oral health goal for 12-year-olds. *International Dental Journal*.

[15] Almerich-Silla J. M., Montiel-Company J. M., Ruiz-Miravet A. (2008). Caries and dental fluorosis in a western Saharan population of refugee children. *European Journal of Oral Sciences*.

[16] Petersen P. E., Kaka M. (1999). Oral health status of children and adults in the Republic of Niger, Africa. *International Dental Journal*.

[17] Abid A. (2004). Oral health in Tunisia. *International Dental Journal*.

[18] Abid A., Maatouk F., Berrezouga L. (2015). Prevalence and severity of oral diseases in the Africa and Middle East Region. *Advances in Dental Research*.

[19] Adekoya-Sofowora C. A., Nasir W. O., Oginni A. O., Taiwo M. (2006). Dental caries in 12 year old suburban Nigerian school children. *African Health Sciences*.

[20] Varenne B., Petersen P. E., Ouattara S. (2004). Oral health status of children and adults in urban and rural areas of Burkina Faso, Africa. *International Dental Journal*.

[21] Mazza C., Strohmenger L., Campus G., Cagetti G., Caruso F., Petersen P. E. (2010). Oral health status of children living in Gorom-Gorom, Oudalan District, Burkina Faso. *International Journal of Dentistry*.

[22] Beiruti N., Taifour D., van Palenstein-Helderman W. H., Frencken J. E. (2001). A review of the oral health status in Syria. *International Dental Journal*.

[23] Beiruti N., van Palenstein-Helderman W. H. (2004). Oral health in Syria. *International Dental Journal*.

[24] Dashash M., Blinkhorn A. (2012). The dental health of 5 year-old children living in Damascus, Syria. *Community Dental Health*.

[25] Paul T. R. (2003). Dental health status and caries pattern of preschool children in Al-Kharj, Saudi Arabia. *Saudi Medical Journal*.

[26] Hashim R., Williams S. M., Thomson W. M., Awad M. A. (2010). Caries prevalence and intra-oral pattern among young children in Ajman. *Community Dental Health*.

[27] Sayegh A., Dini E. L., Holt R. D., Bedi R. (2005). Oral health, sociodemographic factors, dietary and oral hygiene practices in Jordanian children. *Journal of Dentistry*.

[28] Bravo M., Cortés F. J., Casals E., Llena C., Almerich-Silla J. M., Cuenca E. (2009). Basic oral health goals for Spain 2015/2020. *International Dental Journal*.

[29] Saltaji H., Alfakir H. (2015). Oral health consequences of the crisis in Syria. *British Dental Journal*.

[30] UNHCR (2006). *2005 Global Refugee Trends. Statistical Overview of Populations of Refugees, Asylum-Seekers, Internally Displaced Persons, Stateless Persons, and Other Persons of Concern to UNHCR*.

[31] Petersen P. E. (2004). Challenges to improvement of oral health in the 21st century–the approach of the WHO Global Oral Health Programme. *International Dental Journal*.

[32] Petersen P. E. (2005). Sociobehavioural risk factors in dental caries–international perspectives. *Community Dentistry and Oral Epidemiology*.

[33] Fejerskov O., Larsen M. J., Richards A., Baelum V. (1994). Dental tissue effects of fluoride. *Advances in Dental Research*.

[34] Amin T. T., Al-Abad B. M. (2008). Oral hygiene practices, dental knowledge, dietary habits and their relation to caries among male primary school children in Al Hassa, Saudi Arabia. *International Journal of Dental Hygiene*.

[35] Baelum V., Scheutz F. (2002). Periodontal diseases in Europe. *Periodontol 2000*.

